# Atypical florid vascular proliferation in appendix: a diagnostic dilemma

**DOI:** 10.1186/1746-1596-8-12

**Published:** 2013-01-24

**Authors:** Mi Jin Gu, Joon Hyuk Choi, So Hyun Kim

**Affiliations:** 1 Department of Pathology, Yeungnam University College of Medicine, 170 Hyeonchung-ro, Nam-gu, Daegu, Rep of Korea; 2 Department of Surgery, Yeungnam University College of Medicine, 170 Hyeonchung-ro, Nam-gu, Daegu, Rep of Korea

**Keywords:** Blood vessel, Proliferation, Atypical, Appendix

## Abstract

Vascular abnormality of the intestine is rare, except angiodysplasia. We report on an unusual case of atypical florid vascular proliferations of the appendix. A 41-year old male presented with melena. Adhesioned blood clots in the appendiceal orifice were observed by colonoscopy. He underwent laparoscopic appendectomy. Microscopically, a tiny exophytic polypoid mass was observed. The mass showed pyogenic granuloma-like features in the superficial portion and infiltrative florid vascular proliferations in the deeper portion. Endothelial cells showed minimal nuclear atypia, and mitotic figures were observed infrequently and showed positivity for CD31 and CD34 and negativity for HHV-8. Differential diagnoses include from benign vascular tumor to angiosarcoma or Kaposi’s sarcoma, but this lesion does not fit the description of any defined vascular entity. We diagnosed atypical florid vascular proliferations and the patient has been well during the five-month postoperative follow-up.

**Virtual Slide:**

The virtual slide(s) for this article can be found here: http://www.diagnosticpathology.diagnomx.eu/vs/1386921325843104

## Background

Vascular proliferative lesions occurring in the gastrointestinal tract include tumors and tumor-like lesions of the vessels. With the exception of angiodysplasia, vascular abnormalities of the gastrointestinal tract are uncommon [1]. Seven cases of florid vascular proliferations occurring in the intestinal tract have been reported in English literature [1-3]. All reported cases were associated with intussusception or mucosal prolapse and showed microscopic features that overlap with those of angiosarcoma. Florid vascular proliferations are thought to be a secondary response to repeated mucosal trauma and ischemia and differential diagnosis from angiosarcoma is necessary [1]. We herein report on a challenging case showing unusual microscopic findings of the appendix.

## Case report

**Figure 1:**
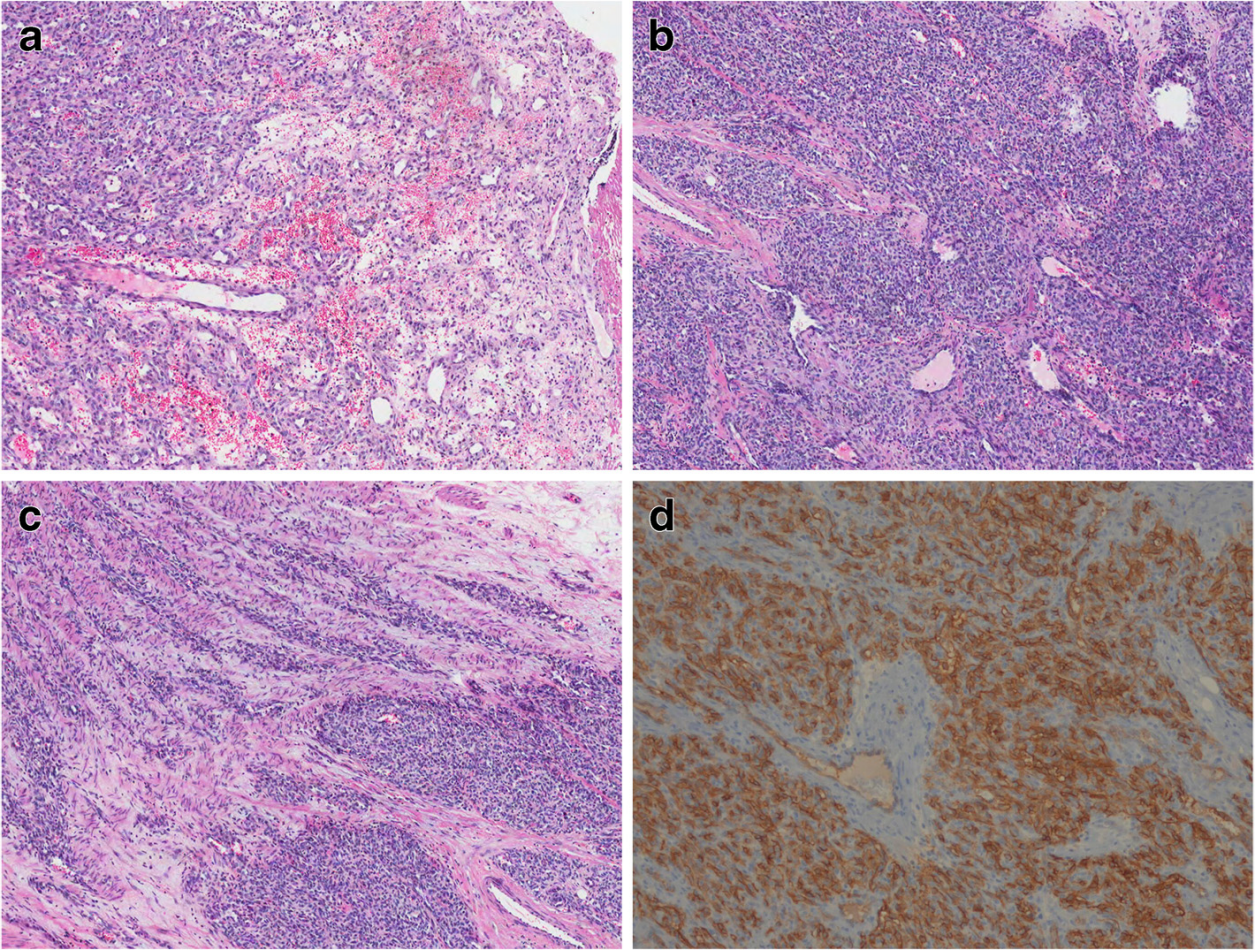
**Morphology: (a) The superficial portion showed pyogenic granuloma-like findings on H&E (original magnification x 100). **(**b**) Endothelial cells maintained vague lobular architectures and exhibited bland cellular morphology on H&E (original magnification x 40). (**c**) The deeper portion showed infiltrative growth into the muscularis propria on H&E (original magnification x 40). (**d**) Endothelial cells showed a positive result for CD34 (original magnification x 100).

A 41-year old male presented with melena. He had no other symptoms, such as migratory pain, right lower quadrant abdominal tenderness with guarding, or leukocytosis. Colonoscopy showed non-specific findings, except for adhesioned blood clots in the appendiceal orifice. He underwent laparoscopic appendectomy. No characteristic features were observed on the external surface. Cut sections showed a tiny polypoid mucosa with easy contact bleeding and the appendiceal lumen was filled with blood. Microscopically, inflammatory cells infiltration, diverticulum, parasite, and fecalith were not observed. An exophytic polypoid mass with extensive surface ulceration was observed. The superficial portion of the polyp showed pyogenic granuloma-like features and the deeper portion was composed of vaguely lobulated proliferation of closely packed small capillary-sized vessels and showed infiltrative growth and extension into muscularis propria (Figure 1). Endothelial cells had round to ovoid nuclei and showed minimal nuclear atypia without multi-layering. Mitotic figures were observed infrequently. Endothelial cells stained for CD31 and CD34, but did not stain for human herpes virus type 8 (HHV-8). Immunostaining for SMA, as well as a reticulin stain, fails to demonstrate a convincing well-formed vascular architecture of the type seen in capillary hemangioma; these small vessels are not surrounded by the usual well-developed layer of pericytes and reticulin fibers. Ki-67 labeling index was less than 5% and p53 positive endothelial cells were observed rarely. The above mentioned microscopic findings do not fit the description of any defined vascular tumor entity.

## Discussion

In our review of the English literature, vascular tumors or vascular lesions occurring in the appendix have been rarely reported [4-9]. This lesion exhibited infiltrative growth with bland endothelial cell morphology; therefore, we were faced with a dilemma.

Differential diagnoses include from reactive response to preceding appendicitis, reactive vascular lesion, benign to malignant vascular tumor. However, this lesion did not fit the description of any defined benign vascular tumor entity. For reactive response to preceding appendicitis, he had no previous history of appendicitis and there were no microscopic features of acute or chronic appendicitis. The florid benign vascular proliferations occurring in the gastrointestinal tract have been reported and may be confused with angiosarcoma [1-3]. The lesions showed proliferation of small vascular channels extending through the bowel wall, similar to angiosarcoma. However, these lesions maintained lobular architecture and did not feature dissecting, atypical vascular channels with plump and multilayered endothelial cells with nuclear atypia [10]. All of the reported cases of florid vascular proliferations were associated with intussusception and mucosal prolapsed; such clinical findings were not observed in this case. However, in this case, similar microscopic features with florid vascular proliferations were observed. Kaposi’s sarcoma, a unique mesenchymal tumor of blood and lymphatic vessels, and is associated with acquired immune deficiency syndrome (AIDS) [9,11-13]. Kaposi’s sarcoma may involve any organs and the gastrointestinal tract is the third most affected site. In this case, there was no proliferation of spindle cells with atypical nuclei, producing small slit-like lumina within vascular spaces containing red blood cells, and the patient was a human immunodeficiency virus (HIV)-negative heterosexual man.

Angiosarcomas account for less than 1% of all soft tissue sarcomas [10,14]. Primary gastrointestinal angiosarcoma is exceedingly rare and usually involves stomach and small bowel. Gastrointestinal angiosarcoma usually presents with gastrointestinal bleeding and anemia [10]. Although microscopic findings are differed from those of angiosarcoma, we could not exclude the possibility of a deceptively bland metastasis or primary angiosarcoma which closely mimicks hemangioma. On further work-up, no abnormal lesion was observed in any other organ. The patient has been well during the five-month postoperative follow-up.

In summary, based on the histologic and clinical findings, we think that this was case of atypical florid vascular proliferatons, rather than angiosarcoma. Consideration of this lesion may helpful in to prevention of misdiagnosis.

## Consent

Written informed consent was obtained from the patient described in this case report and any accompanying images. A copy of the written consent is available for review by the Editor-in Chief of this journal.

## Competing interests

The authors declare that they have no competing interests.

## Authors’ contributions

MJG designed the study, drafted the manuscript and approved of the version to be published. SHK collected clinical data. JHC revised for important intellectual content. All authors read and approved the final manuscript.
